# Antenatal exposure to solar radiation and learning disabilities: Population cohort study of 422,512 children

**DOI:** 10.1038/s41598-019-45562-9

**Published:** 2019-06-27

**Authors:** Claire E. Hastie, Daniel F. Mackay, Tom L. Clemens, Mark P. C. Cherrie, Albert King, Chris Dibben, Jill P. Pell

**Affiliations:** 1 Institute of Health and Wellbeing, University of Glasgow, Glasgow, G12 8RZ UK; 2 Centre for Research on Environment, Society and Health, School of Geosciences, University of Edinburgh, Edinburgh, Scotland, EH8 9XP UK; 3 ScotXed, Scottish Government, Edinburgh, EH6 6QQ UK; 4 Institute of Geography, Drummond Street, University of Edinburgh, Edinburgh, EH8 9XP UK

**Keywords:** Risk factors, Environmental impact, Epidemiology, Paediatric research

## Abstract

Learning disability varies by month of conception. The underlying mechanism is unknown but vitamin D, necessary for normal brain development, is commonly deficient over winter in high latitude countries due to insufficient ultraviolet radiation. We linked the 2007–2016 Scottish School Pupil Censuses to Scottish maternity records and to sunshine hours and antenatal ultraviolet A/B radiation exposure derived from weather stations and satellites respectively. Logistic regression analyses were used to explore the associations between solar radiation, then ultraviolet B, and learning disabilities, adjusting for the potential confounding effects of month of conception and sex. Of the 422,512 eligible, singleton schoolchildren born at term in Scotland, 79,616 (18.8%) had a learning disability. Total antenatal sunshine hours (highest quintile; adjusted OR 0.89; 95% CI: 0.86, 0.93; p < 0.001) and ultraviolet B exposure (highest quintile; adjusted OR 0.55; 95% CI: 0.51, 0.60; p < 0.001) were inversely associated with learning disabilities with evidence of a dose-relationship. The latter association was independent of ultraviolet A exposure. Significant associations were demonstrated for exposure in all three trimesters. Low maternal exposure to ultraviolet B radiation may play a role in the seasonal patterning of learning disabilities. Further studies are required to corroborate findings and determine the effectiveness of supplements.

## Introduction

Learning disabilities have profound, long-term effects on affected individuals, their families and society as a whole. The lifetime cost of affected children born over one year in the USA has been estimated at over $51 billion^1^. Identifying and addressing modifiable risk factors could reduce the burden on health and social care. We previously demonstrated seasonal patterning whereby the risk of learning disabilities varied by month of conception;^2^ with seasonality accounting for 11.4% of all cases of learning disabilities.

During the antenatal period, the fetus undergoes rapid development and growth making it susceptible to environmental exposures with the potential of long-term consequences.

Maternal serum concentrations of vitamin D are important for normal brain development^3^. Exposure to UVB radiation promotes vitamin D production. As a result of low levels of UVB radiation, vitamin D deficiency is common over winter months in high latitude countries^4^. Therefore, seasonal variation in maternal vitamin D concentrations over the critical period of fetal development is a biologically plausible mechanism underlying the seasonal patterning of learning disability.

In order to test this hypothesis, we linked Scotland-wide education and maternity records with satellite data to explore the associations between total, and trimester-specific, sunshine hours and UVB exposure, and learning disability. Sunshine hours are a proxy of total UV radiation but the amount of radiation reaching Earth’s surface varies with solar activity and the solar cycle. Therefore, adjustment of the sunshine hours with sunspot data provides a better proxy of women’s total UV exposure. UV levels are strongly correlated with season; however, superimposed on this annual cycle, there is an 11-year solar cycle over which changes in the sun’s oscillatory magnetic field result in variations in the number of sunspots and, therefore, solar radiation. As a result, UV levels vary between years and their associations can be disentangled from other seasonally patterned phenomena such as temperature and lifestyle by adjusting for month of conception.

Furthermore, whilst UVA radiation covers longer wavelengths and is not absorbed by the ozone layer, UVB radiation covers shorter wavelengths and, therefore, is mostly absorbed before reaching the earth’s surface^5^. The actual amount of UVB reaching the earth’s surface varies greatly with weather conditions, such as cloud cover^6^. Therefore, whilst levels of UVA and UVB are correlated, there is sufficient variation to determine if associations with solar radiation are driven by UVA or UVB.

We explored whether overall UV radiation (sunshine hours adjusted for sunspots) was associated with learning disabilities before examining UVB specifically.

## Results

Overall, 1,023,592 singleton children who attended Scottish schools at some point between 2007 and 2016 were born in Scotland. Of these, 601,066 were ineligible for inclusion: 57,687 were born <37 weeks gestation; 38,010 were born >41 weeks gestation, 1,058 had missing data on gestation; 72 had a birthweight <400 g; 279 had a birthweight >6,500 g; one was <4 years old; four were >19 years old; 486,403 were conceived prior to 2000 or born after 2011; and 17,552 could not be linked to sunshine hours data. The remaining 422,526 were eligible for inclusion. A further 14 had to be excluded because the child’s sex was missing. Therefore, the final study population comprised 422,512 schoolchildren. Of these 79,616 (18.8%) had a learning disability: 49,770 (23.1%) boys and 29,846 (14.4%) girls.

**Figure 1:**
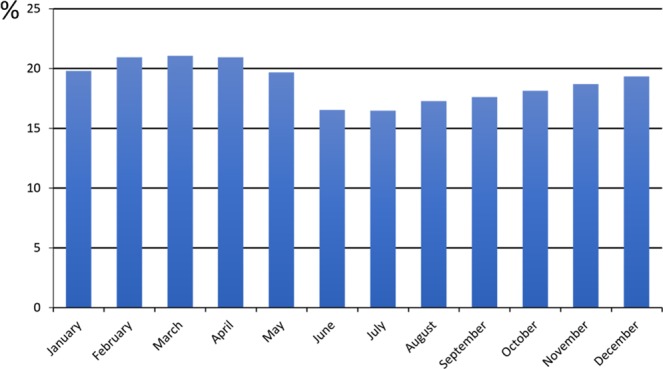
Crude prevalence of learning disability by month of conception.

The crude prevalence of children with learning disabilities varied by month of conception; ranging from 16.5% among children conceived in July to 21.0% among those conceived in February, March and April (Fig. 1). Over the 11 year study period (2000–2010), mean exposure to UVB ranged from 0.50 kJ/m^2^ (range 0.40 kJ/m^2^ to 0.62 kJ/m^2^) in December to 19.84 kJ/m^2^ (range 17.93 kJ/m^2^ to 20.01 kJ/m^2^) in June.

After adjusting for month of conception, there was a statistically significant inverse association between the total sunshine hours over the whole pregnancy and learning disabilities that persisted after adjusting for the child’s sex and the number of sunspots (Table 1). The trimester-specific analyses only reached statistical significance for sunshine hours in trimester 1. Following adjustment for month of conception, there was also a statistically significant, dose-dependent inverse relationship between UVB exposure over the whole of pregnancy and risk of learning disability (Table 2). This persisted after adjustment for the child’s sex and was independent of UVA exposure. The associations were significant for exposure in all three trimesters and persisted after adjustment for UVB exposure in the other two trimesters (Table 2).

**Table 1:** Associations Between Quintile of Overall and Trimester-specific Number of Sunshine Hours and Learning Disability, Scotland, 2000–2010.

		Trimester 1		Trimester 2		Trimester 3		Whole pregnancy	
		OR (95% CI)	P value	OR (95% CI)	P value	OR (95% CI)	P value	OR (95% CI)	P value
Univariate	1 (lowest)	Referent		Referent		Referent		Referent	
	2	0.92 (0.90–0.94)	<0.01	1.06 (1.03–1.09)	<0.01	0.97 (0.94–0.99)	0.01	1.01 (0.99–1.04)	0.45
	3	0.94 (0.91–0.96)	<0.01	1.26 (1.23–1.29)	<0.01	1.00 (0.97–1.02)	0.69	1.10 (1.07–1.13)	<0.01
	4	0.89 (0.86–0.91)	<0.01	1.24 (1.21–1.27)	<0.01	1.06 (1.03–1.08)	<0.01	1.15 (1.12–1.18)	<0.01
	5 (highest)	0.85 (0.83–0.87)	<0.01	1.19 (1.16–1.22)	<0.01	1.11 (1.09–1.14)	<0.01	1.10 (1.07–1.12)	<0.01
Multivariate*	1 (lowest)	Referent		Referent		Referent		Referent	
	2	0.93 (0.90–0.96)	<0.01	1.02 (0.98–1.05)	0.37	0.97 (0.94–1.01)	0.09	0.96 (0.94–0.99)	0.01
	3	0.92 (0.88–0.96)	<0.01	1.10 (1.05–1.16)	<0.01	1.03 (0.98–1.08)	0.19	0.97 (0.94–1.00)	0.02
	4	0.86 (0.81–0.90)	<0.01	1.05 (1.00–1.11)	0.07	1.03 (0.98–1.09)	0.22	0.93 (0.90–0.97)	<0.01
	5 (highest)	0.82 (0.77–0.87)	<0.01	1.00 (0.95–1.06)	0.96	1.02 (0.97–1.08)	0.45	0.84 (0.81–0.87)	<0.01
Multivariate**	1 (lowest)	Referent		Referent		Referent		Referent	
	2	0.92 (0.89–0.96)	<0.01	1.02 (0.98–1.05)	0.35	0.97 (0.94–1.00)	0.07	0.96 (0.94–0.99)	<0.01
	3	0.92 (0.88–0.96)	<0.01	1.11 (1.05–1.16)	<0.01	1.03 (0.99–1.08)	0.19	0.97 (0.94–0.99)	0.02
	4	0.86 (0.81–0.90)	<0.01	1.05 (1.00–1.11)	0.06	1.03 (0.98–1.09)	0.22	0.93 (0.90–0.96)	<0.01
	5 (highest)	0.81 (0.77–0.86)	<0.01	1.00 (0.95–1.06)	0.92	1.02 (0.96–1.08)	0.51	0.84 (0.81–0.87)	<0.01
Multivariate***	1 (lowest)	Referent		Referent		Referent		Referent	
	2	0.93 (0.91–0.96)	<0.01	1.01 (0.98–1.05)	0.50	0.95 (0.91–0.98)	<0.01	0.97 (0.94–1.00)	0.02
	3	0.88 (0.84–0.92)	<0.01	1.01 (0.96–1.06)	0.61	0.93 (0.89–0.97)	<0.01	0.97 (0.94–1.00)	0.04
	4	0.85 (0.81–0.90)	<0.01	0.96 (0.91–1.02)	0.17	0.94 (0.89–0.99)	0.02	0.95 (0.92–0.98)	0.01
	5 (highest)	0.86 (0.81–0.91)	<0.01	0.97 (0.91–1.03)	0.26	0.95 (0.89–1.00)	0.06	0.89 (0.86–0.93)	<0.01
Multivariate****	1 (lowest)	Referent		Referent		Referent			
	2	0.93 (0.90–0.96)	<0.01	1.01 (0.98–1.05)	0.57	0.95 (0.92–0.98)	<0.01		
	3	0.88 (0.84–0.92)	<0.01	1.01 (0.96–1.06)	0.83	0.93 (0.89–0.98)	<0.01		
	4	0.85 (0.80–0.90)	<0.01	0.96 (0.91–1.02)	0.15	0.93 (0.89–0.99)	0.01		
	5 *highest	0.85 (0.80–0.90)	<0.01	0.98 (0.92–1.04)	0.43	0.95 (0.90–1.01)	0.11		

CI confidence interval; OR odds ratio.

*Adjusted for month of conception; **also adjusted for child’s sex; ***also adjusted for sun spots; ****also adjusted for sun hours in other trimesters.

**Table 2:** Associations Between Quintile of Overall and Trimester-specific Antenatal Exposure to UVB and Learning Disability, Scotland, 2000–2010.

		Trimester 1		Trimester 2		Trimester 3		Whole pregnancy	
		OR (95% CI)	P value	OR (95% CI)	P value	OR (95% CI)	P value	OR (95% CI)	P value
Univariate	1 (lowest)	Referent		Referent		Referent		Referent	
	2	0.98 (0.96–1.01)	0.16	1.00 (0.98–1.03)	0.96	1.05 (1.02–1.07)	<0.01	1.03 (0.98–1.03)	0.80
	3	1.02 (1.00–1.05)	0.07	1.12 (1.10–1.15)	<0.01	1.07 (1.04–1.09)	<0.01	1.09 (1.06–1.12)	<0.01
	4	1.02 (1.00–1.05)	0.06	1.23 (1.20–1.26)	<0.01	1.03 (1.00–1.05)	0.04	1.21 (1.18–1.24)	<0.01
	5 (highest)	0.95 (0.93–0.97)	<0.01	1.20 (1.17–1.23)	<0.01	0.99 (0.96–1.01)	0.26	1.18 (1.15–1.21)	<0.01
Multivariate*	1 (lowest)	Referent		Referent		Referent		Referent	
	2	0.98 (0.93–1.04)	0.50	0.96 (0.91–1.02)	0.16	1.14 (1.08–1.20)	<0.01	0.90 (0.86–0.95)	<0.01
	3	0.98 (0.91–1.07)	0.67	0.79 (0.72–0.87)	<0.01	1.33 (1.21–1.47)	<0.01	0.75 (0.70–0.80)	<0.01
	4	0.77 (0.70–0.86)	<0.01	0.65 (0.58–0.72)	<0.01	0.96 (0.86–1.080)	0.52	0.64 (0.59–0.69)	<0.01
	5 (highest)	0.58 (0.52–0.65)	<0.01	0.42 (0.37–0.48)	<0.01	0.48 (0.42–0.55)	<0.01	0.56 (0.51–0.61)	<0.01
Multivariate**	1 (lowest)	Referent		Referent		Referent		Referent	
	2	0.98 (0.93–1.04)	0.47	0.96 (0.91–1.02)	0.15	1.14 (1.08–1.20)	<0.01	0.90 (0.86–0.95)	<0.01
	3	0.98 (0.90–1.06)	0.55	0.79 (0.72–0.86)	<0.01	1.34 (1.21–1.48)	<0.01	0.74 (0.69–0.79)	<0.01
	4	0.77 (0.69–0.85)	<0.01	0.64 (0.57–0.72)	<0.01	0.96 (0.86–1.07)	0.47	0.64 (0.59–0.69)	<0.01
	5 (highest)	0.57 (0.51–0.64)	<0.01	0.42 (0.37–0.47)	<0.01	0.48 (0.41–0.54)	<0.01	0.55 (0.51–0.60)	<0.01
Multivariate***	1 (lowest)	Referent		Referent		Referent		Referent	
	2	0.95 (0.89–1.01)	0.10	0.91 (0.85–0.98)	0.01	1.04 (0.98–1.11)	0.24	0.84 (0.80–0.88)	<0.01
	3	0.61 (0.54–0.68)	<0.01	0.67 (0.60–0.74)	<0.01	1.14 (1.03–1.27)	0.02	0.61 (0.57–0.66)	<0.01
	4	0.43 (0.38–0.50)	<0.01	0.50 (0.44–0.56)	<0.01	0.86 (0.76–0.97)	0.01	0.55 (0.51–0.60)	<0.01
	5 (highest)	0.34 (0.30–0.40)	<0.01	0.34 (0.30–0.39)	<0.01	0.45 (0.39–0.52)	<0.01	0.50 (0.46–0.55)	<0.01
Multivariate****	1 (lowest)	Referent		Referent		Referent			
	2	0.88 (0.82–0.94)	<0.01	0.96 (0.90–1.03)	0.26	1.06 (1.00–1.13)	0.07		
	3	0.64 (0.57–0.73)	<0.01	0.70 (0.63–0.78)	<0.01	0.96 (0.84–1.09)	0.51		
	4	0.44 (0.37–0.51)	<0.01	0.52 (0.46–0.59)	<0.01	0.63 (0.54–0.73)	<0.01		
	5 *highest	0.38 (0.32–0.44)	<0.01	0.36 (0.32–0.42)	<0.01	0.33 (0.28–0.39)	<0.01		

CI confidence interval; OR odds ratio.

*Adjusted for month of conception; **also adjusted for child’s sex; ***also adjusted for UVA; ****also adjusted for UVB in other trimesters.

## Discussion

Among 422,512 children attending Scottish schools over a ten-year period, antenatal exposure to UVB was inversely associated with the risk of learning disability, with some evidence of a dose relationship, and independent of other seasonally patterned phenomena, the child’s sex and UVA exposure. Most vitamin D results from production in the skin following exposure to UVB. Because of its shorter wavelength UVB, unlike UVA, can be absorbed before reaching the earth’s surface. The amount of UVB reaching the earth’s surface varies by both season and weather conditions. In high latitude countries, such as Scotland, the amount of UVB reaching the earth’s surface in winter months is generally insufficient for vitamin D production. Hence, vitamin D insufficiency, as defined by circulating 25-hydroxy vitamin D concentrations below 40 nmol/l, is twice as likely to occur in Scottish residents, compared with other parts of Britain^7^. In both European and Oceanic countries, expression of the nuclear vitamin D receptor has been shown to be seasonally patterned, with peak expression in summer^8^. Residents of Iceland, where there is 24 h daylight in summer and 24 h darkness in winter, do not display the same seasonal patterning of gene expression, suggesting a photoperiod effect^9^.

Low concentrations of maternal vitamin D have been associated with changes in brain size and morphology^10^. Animal models have demonstrated adult brain dysfunction and hyperlocomotion among the offspring of animals deficient in vitamin D in pregnancy^11^. Whitehouse *et al*. measured 25-dihydroxyvitamin D concentrations in 743 white mothers at 18 weeks gestation and found an association with language impairment in their offspring at 5 and 10 years of age^12^.

Low exposure to UV radiation has been postulated as an explanation for the higher incidence of autism in high latitude countries, urban areas, and dark-skinned people^3^. Two recent analyses of the Generation R Cohort in the Netherlands demonstrated associations between gestational vitamin D deficiency and autism spectrum disorder^13^ and autism-related traits^14^. These findings are supported by review articles showing inverse associations between gestational vitamin D and more general neurodevelopment^15,16^. Furthermore, there is evidence of an inverse relationship between 25-hydroxyvitamin D measured in neonatal dried blood samples and risk of both autistic spectrum disorder^17,18^ and schizophrenia^19^. Intervention studies conducted in early childhood have shown that administration of multivitamins containing vitamin D can reduce symptoms in children with autistic spectrum disorder^20^, and improve normal childhood cognition^21^. To date, no studies have investigated whether vitamin D supplements can reduce seasonal patterning in autism or other causes of learning disability.

The marginally stronger effect size observed in trimester 1 in the current analysis suggests that this period of pregnancy may be most vulnerable to the effects of insufficient solar radiation. During trimester 1, dopamine neurons first develop in the human brain. Our findings are in line with those from studies of vitamin D deficiency in animal models, which support the role of vitamin D in dopamine neuronal development and signalling^22,23^.

Ours was a large-scale, non-selective study covering the whole of Scotland. We used existing health and education databases, but these are subjected to regular quality-assurance checks. The School Pupil Census does not include private schools but, in Scotland, fewer than 5% of children attend private schools. The School Pupil Census does cover both primary and secondary schools and includes mainstream schools, special schools, and special classes/units attached to mainstream schools. Special schools are specifically designed to provide education to children with profound and complex disabilities whose needs cannot be met in mainstream schools and, according to data from the 2011 Scottish Pupil Census, 99% of children with learning disabilities aged 5–16 years are in some form of education. Also, both schools and local authorities have a statutory duty to identify children with special educational needs, provide support, and review its provision. Children on long-term illness absence are also included in the School Pupil Census. Therefore, high ascertainment and recording is likely. We previously undertook a validation study of linkage of the School Pupil Censuses and Scottish Morbidity Records 02 and demonstrated that more than 99% of the linkages made were to the correct maternity record for singleton children^24^.

Due to differences in gestation at delivery, children born on the same date may have had the same trimester in a different season. Therefore, use of month of conception as a covariate, rather than month of delivery, was a strength of this study. UV is not the only exposure that exhibits seasonal patterning. Other environmental exposures, such as temperature and maternal influenza, vary by season, as do lifestyle behaviours such as diet and physical activity. Therefore, adjustment for month of conception enabled us to control for the effects of other seasonally patterned exposures and take advantage of the solar cycle related variations in UV superimposed on seasonal variations. Boys have a different seasonal pattern to conception from girls; with male conceptions peaking in Autumn and female in Spring^25^. Boys are also more prone to some types of learning disability, such as autism and attention deficit hyperactivity disorder^26,27^. Therefore, adjustment for the child’s sex was a strength.

We calculated mean monthly levels of UVA and UVB for Scotland as a whole. Mainland Scotland measures 441k (274 miles) from north to south; this equates to a relatively small range of latitudes (from 58.4° North to 58.6° North). Our calculation of UV exposure assumed that women resident in Scotland remained in Scotland during their pregnancy. No data were available on changes in UV exposure due to holidays and trips aboard. Similarly, we had no data on individual differences in exposure to UV due to the proportion of time spent outdoors, choice of clothing, or use of sunblock. Finally, whilst our findings are consistent with a possible role of vitamin D, other photo-active compounds may also be relevant.

In conclusion, low UVB exposure in winter months may be contributing to the patterning of learning disability by month of conception. Further investigation is required to corroborate this hypothesis including measurement of serum vitamin D levels in pregnancy cohorts and intervention studies of vitamin D supplements.

## Methods

The School Pupil Census is conducted annually by all local authority run primary, secondary and special schools in Scotland and the data are collated by the Scottish Exchange of Educational Data (ScotXed). The census records whether the child has a special educational need; defined as being unable to benefit fully from school education without help beyond that normally given to schoolchildren of the same age. We defined learning disability as a record of special educational need attributed to: intellectual disabilities, language or speech disorder including dyslexia, physical or motor impairment, autistic spectrum disorder or social, emotional or behavioural difficulties.

We used probabilistic matching to link individual children included in any School Pupil Census conducted between 2007 and 2016 inclusive, to their maternity record (Scottish Morbidity Record 02 (SMR02)). The linkage methodology has been described in detail previously and demonstrated to be 99% accurate for singleton children^24^. The SMR02 records the child’s sex and date of birth, gestation at delivery, and whether it was a multiple delivery. Since the early 1990s, gestational age has been confirmed by ultrasound conducted in the first half of pregnancy in more than 95% of pregnant women in the United Kingdom. If there is more than seven days difference between gestational age calculated from ultrasound and from last menstrual period, the former is used^28^. Date of conception was derived from date of delivery minus gestational age at delivery plus two weeks. Trimester 1 comprised the first three months of pregnancy from month of conception; trimester 2 comprised months four, five, and six; and trimester 3 comprised all subsequent months until delivery.

Monthly mean, 5 km × 5 km gridded, sunshine hours were downloaded from the Meteorological Office^29^. https://www.metoffice.gov.uk/climate/uk/data/ukcp09/faq#faq10. These data are modelled estimates based on measured sunshine values from the network of static sensors (Kipp & Zonen and Campbell-Stokes sensors) across Scotland. Monthly mean sunspot numbers for the northern hemisphere are produced by the Royal Observatory of Belgium in Brussels and were obtained from the Sunspot Index and Long-term Solar Observations (SILSO) website^30^. Global 5 km UV radiation irradiance data were produced by the Japan Aerospace Exploration Agency (JAXA) using measurements from the moderate resolution imaging spectroradiometer (MODIS) instrument onboard NASA’s aqua and terra satellites. Downward irradiance values (i.e. combined direct and diffuse radiation on a horizontal plane) for UVA (315nm-400nm) and UVB (280–315 nm) are available at a daily resolution from 2000 to 2010 inclusive. We downloaded every binary file available from 1 January 2000 to 31 December 2010 from the publicly accessible JAXA FTP site (ftp://apollo.eorc.jaxa.jp/pub/JASMES/Global_05km/). We extracted the contents of each binary file, which gave us a UVA and UVB value for each latitude and longitude on earth. We then deleted any points that were not contained within a Great Britain bounding box (48°N to 63°N; –11°W to 4°E). The Great Britain points were then interpolated, via inverse weighted distance, to raster images. The rasters were projected to Ordnance Survey Great Britain (OSGB) 1936/British National Grid. Finally, we took the Northing and Easting centroids of each postcode unit in Scotland (Ordnance Survey codepoint ver 2017.4.0) and used these to extract the UVA and UVB values from only those grid squares covering residential areas in Scotland. Scotland-wide monthly means were then calculated resulting in a monthly time-series of UVA and UVB irradiance values measured in W/m^2^. These were converted to kJ/m^2^ by multiplying by 86,400 (seconds in the day) and dividing by 1,000 to be consistent with other epidemiological studies. For the four environmental exposures (sunshine hours; number of sunspots; UVA radiation; and UVB radiation) we calculated exposure over each trimester of pregnancy and total exposure over the whole of pregnancy. Each was then categorised into overall and trimester-specific quintiles.

Inclusion in the study was restricted to singleton children born in Scotland at term; defined as 37–41 weeks gestation inclusive. We excluded children whose age was recorded as <4 years or >19 years in the School Pupil Census. Since UV data were only available for 2000–2010 inclusive, children conceived prior to 2000 or born after 2011 were excluded as they did not have UV exposure data for the whole of their intra-uterine period.

Firstly, we tested whether there was evidence of any association between overall solar radiation and learning disabilities using sunshine hours as a proxy measure of the former. A series of univariate logistic regression analyses were performed of the associations between trimester specific and overall sunshine hours and learning disability. The models were then adjusted sequentially for month of conception then child’s sex then number of sunspots. The trimester specific models were then adjusted for the number of sunshine hours in the other two trimesters. Secondly, we explored whether there were associations specific to UVB exposure.

The same models were run using UVB as the exposure and adjusting for month of conception, then child’s sex, then UVA exposure and, finally, the trimester specific models were adjusted for UVB exposure in the other two trimesters. All analyses were undertaken using Stata v14.

## Data Availability

The datasets analysed during the current study are available in the National Services Scotland National Safe Haven, https://www.isdscotland.org/Products-and-Services/eDRIS/Use-of-the-National-Safe-Haven/.
